# Supplementary material for: Learning curve and surgical outcome of robotic assisted colorectal surgery with ERAS program
Source: Sci Rep. 2022 Nov 29;12:20566. doi: 10.1038/s41598-022-24665-w (PMC9709162; doi:10.1038/s41598-022-24665-w)
Supplement: Supplementary file 2 — Supplementary Information 2. [file 41598_2022_24665_MOESM2_ESM.zip › RAL Raw data-Ñ[▒K/095/OP_ñw╝╨░O▒Kñσ.pdf]

正式摘要(電子簽章：000000220637577)

W52 / W52

臺中榮民總醫院

Operation Note

[redacted]

- 手術日期：2021/07/01
- 手術醫師：VS 林俊余 , CR 林陳彥 , CR 陳映菁 , R 郭連雄
- Anesthesia : GE
- Position : Lithotomy
- Aseptic agents : Soap , Betadine Alcohol , Betadine Aqua

Diagnosis

- Pre-operative diagnosis : rectum ca 4 cm 6" s/p SCRT
- Post-operative diagnosis : Ditto.

Surgical Indication

- tumor resection

Operation

- 1. Robotic (XI) assisted TME + loop ileostomy(LLQ)
  2. Intraoperative colonoscopy : (-)
  3. High ligation (+) : At IMA root. Left colic artery and IMV were also ligated
  4. Take down from the splenic flexure : (-)
  5. Robotic endoGIA (45-4.3) X2 + EEA (29mm) were used
  6. Proximal cut end: 11 cm, distal cut end: 1 cm
  7. Penrose drain was inserted from RLQ to pelvis
  8. ICG: proximal 33sec. rectum stump 35sec

Finding

- 1. A 3.5*3cm cm tumor over lower rectum
  2. No other gross tumor nor polyps was found
  3. There was no obvious visible liver metastasis, no visible peritoneal seeding
  4. No obvious ascites was noted
  5. Bilateral doughnuts : intact ; Air leak test: Done and no air leak was found

Procedure

- 1. General endotracheal anesthesia was induced and then the patient was placed in modified Trendelenburg lithotomy position.
  2. Preoperative antibiotics were given. A Foley catheter and nasogastric tube were placed.
  3. The abdomen was prepped and draped in standard sterile fashion.
  4. A small vertical incision was made above the umbilicus and a 8 mm trocar was inserted into the peritoneal cavity by open method. Peumoperitoneum was established with CO2 inflation.
  5. Laparoscopy was inserted and then other two 8mm trocars were inserted over LUQ, RLQ of abdomen. Another 12mm trocars was inserted over RLQ area, and the other assist-port 12mm troca was inserted on RUQ. The operative findings were mentioned as above.
  6. The sigmoid colon was held by grasper and the peritoneum overlying the mesentery was scored with electrocautery.
  7. The inferior mesentery artery was identified, ligated above the left colic artery with endoclips, and transected.
  8. Dissection was done above the Gerota's fascia and toward the peritoneal attachments.
  9. The colon was mobilized from retroperitoneum overlying the sacral promontory and into the pelvis. The incision was extended laterally and medially across the peritoneal reflection.
  10. The mesorectum was then mobilized.

- 11. Distal descending colon and rectum were mobilized.
  12. The mesorectum was then mobilized using sharp dissection until the levator ani muscle was identified.
  13. ICG was infused to check blood supply under firefly mode.
  14. The rectum was divided with Robotic endoGIA (45-4.1) X2.
  15. An hornizontal incision was made over LLQ, and the rectum was pulled out.
  16. ICG was infused again to check blood supply under firefly mode.
  17. The proximal colon was then taken out for carefully inspection, and to make sure it has an excellent blood supply. The
  mesenteric fat was gently cleared from the terminal 2-3 mm of the bowel ends.
  18. The field was isolated with moist pad and the bowel opened and sized. It was determined that a 29 mm circular stapler could be accommodated. A purse string suture of 2-0 prolene was placed. The tumor-containing tissue was removed. Then the anvil was inserted, followed by the purse string suture. The colon was then put back to the abdominal cavity.
  19. Pneumoperitoneum was constructed again.
  20. The anal canal was irrigated with distil water and then the circular stapler was introduced through the anus and pierced through the upper area (The ventral site of the body) of the stapler line.
  21. The proximal bowel with anvil in place was engaged with the circular stapler and the stapler was then fired. Two complete donuts were obtained and the distal one was submitted to pathology for margin confirmation.
  22. The anastomosis was tested for leaks with irrigation solution and air introduced through the rectum and was found to be patent and intact.
  23. The desired ileum site (About 15~20cm from IC valve) was selected for further ostomy construction.
  24. The loop ileostomy was pulled out from LLQ incision wound.
  25. The ileum was retracted from the desired ostomy site. A window was created under the ileum by dividing the mesentery adjacent to the bowel wall with electrical cautery carefully, with a plastic bridge in placed.
  26. Penrose drain was inserted through trocar incision at RLQ and placed into pelvis. The fascia of 12mm trochar sites and central extended wound were closed with interrupted sutures via Num. 1 Vicryl.
  27. The wound was further closed with 3-O Nylon.
  28. Loop ileostomy was matured by 4-0 monocryl. And the lumen of patency was confirmed. Ostomy bag was applied.
  29. The patient tolerated the procedure well and was brought to the POR in stable condition.
- Estimated blood loss: minimum
- 器械數量: 正確
- 紗布數量: 正確

Image

- PAINT
  - colon & rectum

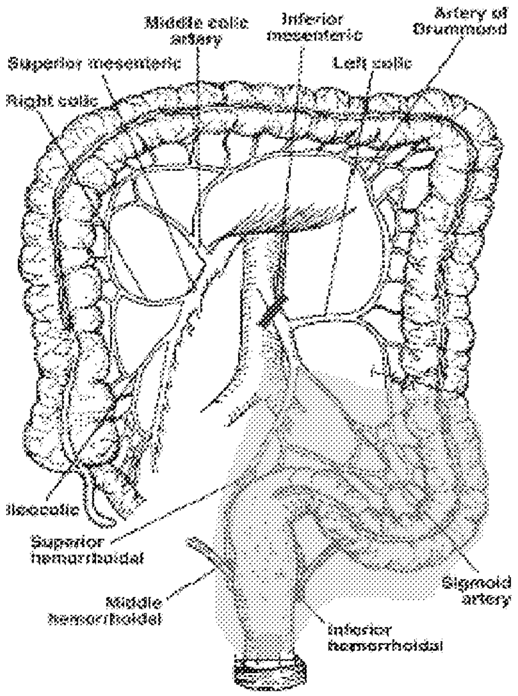

- abdomen

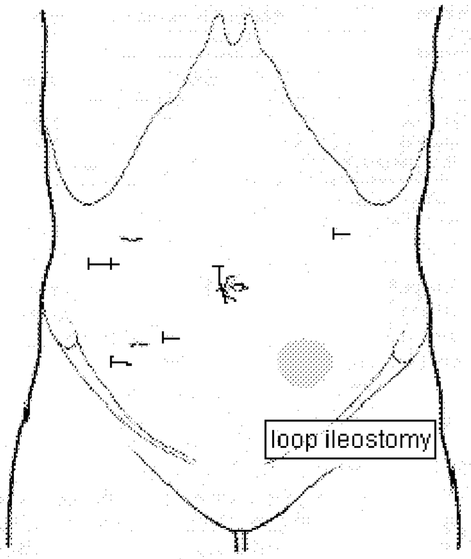

主治 2222H林俊余 / 醫師 2303K陳映菁

正式摘要**(**電子簽章：**000000220637577)**

W52 / W52

doctorRank:B
